# RNA adenosine modifications related to prognosis and immune infiltration in osteosarcoma

**DOI:** 10.1186/s12967-022-03415-6

**Published:** 2022-05-14

**Authors:** Shijie Chen, Jin Zeng, Liping Huang, Yi Peng, Zuyun Yan, Aiqian Zhang, Xingping Zhao, Jun Li, Ziting Zhou, Sidan Wang, Shengyu Jing, Minghua Hu, Yuezhan Li, Dong Wang, Weiguo Wang, Haiyang Yu, Jinglei Miao, Jinsong Li, Youwen Deng, Yusheng Li, Tang Liu, Dabao Xu

**Affiliations:** 1 Department of Spine Surgery, The Third Xiangya Hospital of Central South University, 138 Tongzipo Rd, Changsha, 410013 Hunan China; 2 Department of Obstetrics and Gynecology, The Third Xiangya Hospital of Central South University, 138, Tongzipo Road, Changsha, 410013 China; 3 Shanghai Key Laboratory of Regulatory Biology, Institute of Biomedical Sciences and School of Life Sciences, East China Normal University, 500 Dongchuan Rd, Shanghai, 200241 China; 4 Department of Orthopaedics, The Second Xiangya Hospital of Central South University, 139 Renmin Middle Rd, Changsha, 410011 Hunan China; 5 Department of Anatomy, Histology, and Embryology, Changsha Medical University, 1501 Leifeng Avenue, Changsha, 410219 Hunan China; 6 Department of Orthopedics, The Second Affiliated Hospital of Anhui Medical University, 678 Furong Rd, Hefei, 230601 Anhui China; 7 Department of Orthopeadics, Xiangya Hospital, Central South University, 87 Xiangya Rd, Changsha, 410008 Hunan China; 8 School of Basic Medical Science, Central South University, 172 Tongzipo Rd, Changsha, 410013 Hunan China; 9 The Third Xiangya Hospital of Central South University, 138 Tongzipo Rd, Changsha, 410013 Hunan China

**Keywords:** RNA adenosine modifications, Osteosarcoma, Immune, Prognosis, Drugs

## Abstract

**Background:**

RNA adenosine modifications, which are primarily mediated by “writer” enzymes (RMWs), play a key role in epigenetic regulation in various biological processes, including tumorigenesis. However, the expression and prognostic role of these genes in osteosarcoma (OS) remain unclear.

**Methods:**

Univariate and multivariate Cox analyses were used to construct the RMW signature for OS using Target datasets. RMW expression in OS tissue was detected by qPCR analysis. Xcell and GSVA were used to determine the relationship between RMWs and immune infiltration. The DGIdb and CMap databases were used for drug prediction. In vivo and in vitro experiments showed that strophanthidin elicited antitumor activity against OS.

**Results:**

A 3-RMW (CSTF2, ADAR and WTAP) prognostic signature in OS was constructed using the Target dataset and verified using GEO datasets and 63 independent OS tissues via qPCR analysis. High-risk OS patients had poor overall survival, and the prognostic signature was an independent prognostic factor for OS. Functional studies showed that tumour-, metabolism-, cell cycle- and immune-related pathways were related to high risk. Next, we found that RMW-derived high-risk patients exhibited increased infiltration of M2 macrophages and cDCs. Furthermore, we predicted the potential drugs for OS using the DGIdb and CMap databases. In vivo and in vitro experiments showed that strophanthidin elicited antitumor activity against OS by repressing cell growth and inducing cell cycle arrest at the G1 phase.

**Conclusion:**

The 3-RWM-based prognostic signature established in this study is a novel gene signature associated with immune infiltration, and strophanthidin was identified as a candidate therapy for OS by repressing OS cell growth and the cell cycle.

**Supplementary Information:**

The online version contains supplementary material available at 10.1186/s12967-022-03415-6.

## Introduction

Human osteosarcoma (OS) is the most frequent aggressive bone cancer in children and adolescents [1]. Despite improvements in multimodal therapies, the prognosis of OS remains poor (20–30%) due to the delay in diagnosis and the development of metastasis [2]. Therefore, it is urgent to reveal novel biomarkers and ensure effective OS treatment.

Although RNA modification has been recognized for more than half a century, its cell biology remains largely unexplored [3]. RNA adenosine modification is the most common type of RNA modification, including m^1^A and m^6^A modification. *RNA* editing includes *adenosine*-to-inosine (A-to-I) editing and alternative *polyadenylation* (*APA*) [3]. RNA methylation accounts for approximately 60% of RNA modifications, whereas m^1^A and m^6^A modifications are common and abundant in *RNA* methylation modification by the methylation of the adenine base. These modifications regulate RNA stability and translation [4]. m^1^A and m^6^A modifications play key roles in various cellular processes, thus leading to a variety of diseases, including cancer [5, 6]. Polyadenylation (APA) is a phenomenon in which one gene contains multiple polyadenylation (pA) sites to produce transcript isoforms at either the 3′-untranslated region (UTR) or coding regions [7]. Another common posttranscriptional mechanism is RNA editing, which alters gene expression by regulating the nucleotides of transcripts [8]. The most widespread type of RNA editing is A-to-I, which is catalysed by ADAR enzymes and alters the coding, folding, splicing, or transport of transcripts. Dysregulated A-to-I editing can lead to various diseases, including tumorigenesis [9]. Studies have shown that many of these modifications could be related to a complex network that intersects each other [10]. Recently, these RNA modifications have been reported as biomarkers and play key roles in the progression of tumour prognosis [11, 12]. However, their role in OS remains limited.

In this work, we identified RMW patterns and RNA modification-related DEG patterns that were associated with the prognosis of OS and related to immune infiltration. Next, we constructed and verified an RMW signature of OS, which was not only associated with prognosis but also with immune infiltration and the cell cycle. Finally, strophanthidin was predicted and verified as an effective drug for OS.

## Materials and methods

### Datasets

The expression of OS patients with clinical information was obtained from the GSE21257 and Target datasets. Eighty-five OS patients and 53 OS patients with survival follow-up information were collected from the Target and GSE21257 datasets, respectively.

### Consensus clustering analysis

To determine the RNA modification patterns in OS, 26 RMWs were selected for consensus clustering analysis using the Consensus Cluster Plus package, and k = 2 seemed to be the most appropriate selection.

The DEGs identified between the two RMW patterns were analysed using the “limma” package with adjusted *p* < 0.05. Survival-related DEGs were analysed using univariate Cox regression analysis and subsequently selected for a consensus clustering analysis using the Consensus Cluster Plus package, and k = 2 seemed to be the most appropriate selection.

The survival analysis of patients from the two patterns was analysed using the packages “survminer” and “survival”.

The relationship between patterns and clinical characteristics was analysed using the “RColorBrewer” package.

### The RMW prognostic signature

The survival-related RMWs were analysed using univariate Cox regression analysis. The prognostic signature was analysed using lasso regression and multivariate Cox regression analysis. The risk score was calculated as follows:

Risk score = ∑*i*Coefficient _RMWs_ *Expression _RMWs_.

The OS patients were divided into high- and low-risk groups based on the best cut-off value of the risk score from the TCGA dataset. The R “survival” package was used to assess the relationship between survival and the RMW signature. Univariate and multivariate Cox regressions were used to reveal the independent risk factors for OS using R software. ROC curves were employed to reveal the prognostic value using the R software “ROC” package. The nomogram and calibration curve were analysed using the R “rms” package.

### RMW signature-related pathways

GSEA and GSVA were used to reveal the risk signature-related pathways using the Target dataset. GSEA software (Cambridge, MA, United States) was used to describe the pathways related to RMWs. For GSVA, the signalling pathway alterations between the high- and low-risk groups were analysed using the “GSVA” R package.

### Immune infiltration in OS tissues

The immune infiltration of OS tissues from the Target database was evaluated using xCell (https://xcell.ucsf.edu/). Next, the immune-related signalling and immune cell infiltration in different RMW patterns, DEG patterns and high/low risk groups were analysed using the R “ggpubr” package.

### Clinical samples and qPCR analysis

OS tissues were obtained from 63 OS patients (September 2018 to January 2020) from the Third Xiangya Hospital, Central South University. Our study was approved by the ethics committee of the Third Xiangya Hospital, Central South University.

Total RNA was collected from OS tissues and cells using TRIpure reagent (BioTek, VT, USA) and then reverse transcribed using a HiScript Q RT SuperMix kit (Vazyme, Nanjing, China). Then, qRT–PCR was performed to assess mRNA expression using SYBR Green Master Mix (CWBIO, Jiangsu, China) in an Applied Biosystems QuantStudio 3 Real-Time PCR System (Thermo Fisher Scientific, MA, USA) as previously described [13]. The nucleotide sequences of primers for *CSTF2, ADAR, WTAP* and *β-actin* are listed in Table 1.

**Table 1:** The primers for qPCR

	Forward 5′–3′	Reverse 5′–3′
*β-actin*	CTGTCCCTGTATGCCTCTG	TGATGTCACGCACGATTT
*CSTF2*	CAGCGGTGGATCGTTCTCTAC	AACAACAGGTCCAACCTCAGA
*ADAR*	ATCAGCGGGCTGTTAGAATATG	AAACTCTCGGCCATTGATGAC
*WTAP*	CTTCCCAAGAAGGTTCGATTGA	TCAGACTCTCTTAGGCCAGTTAC

### Drug-Gene Interactions

The Drug-Gene Interaction Database (DGIdb, http://dgidb.genome.wustl.edu/) and CMap database were used to screen drugs that potentially target hub genes in OS [18].

### Cell culture

Human OS cell lines (MG63, HOS and U2OS), L02 and HUVEC cell lines were purchased from American Type Culture Collection (ATCC) (Manassas, VA, USA). All cells were cultured as previously described [14].

### Cell viability assay

The MTT assay was used to detect cell viability. In short, the cells were cultured in 96-well plates overnight and then treated with strophanthidin (0.001 to 100 μM) (Santa Cruz, sc-215914A)/PBS for 24 or 48 h. Then, cell viability was detected using an MTT assay. The IC50 value was calculated using GraphPad Prism.

### Colony-forming assay

OS cells were incubated with strophanthidin for 48 h. Then, ~ 1000 cells were seeded in 6-well plates and cultured for 10 days for the colony forming assay.

### Flow cytometric analysis

OS cells were incubated with strophanthidin for 48 h. Then, flow cytometry was used for the cell cycle assay as previously described [14].

### Western blot analysis

OS cells were incubated with strophanthidin for 48 h. Then, the protein was collected using RIPA buffer with a protease inhibitor cocktail (Roche Applied Science, Indianapolis, USA). The proteins were separated and transferred to PVDF membranes (Millipore, Massachusetts, USA). Primary antibodies against GAPDH (ab8245, Abcam), CDK4 (ab108357, Abcam), CCND1 (ab16663, Abcam) and p53 (ab32389, Abcam) and secondary antibodies were used to detect target proteins.

### Animal experiment

Four- to six-week-old female BALB/c nude mice were maintained in SPF conditions. Nude mice were injected with HOS cells (1 × 10^6^) into the left scapula subcutaneously. For the control group, the mice were administered 200 μl of saline po/day. After 10 days, nude mice were randomly assigned to 2 groups. For the treatment group, mice were treated with strophanthidin (0.5 mg/kg/day). The body weights and the tumour volume were measured every 4 days. After 24 days, all nude mice were sacrificed, and the tumour weights were detected. All animal experiments were approved by the ethics committee of Third Xiangya Hospital, Central South University.

### Statistical analysis

All data from Target and GEO were analysed by R-3.6.1. The differential RMWs were evaluated using the limma and heatmap packages. Student’s *t test* was used for two groups of in vitro experiments, and chi-squared (*χ*^2^) tests were used for in vivo experiments. p < 0.05 was considered statistically significant.

## Results

### RMW patterns associated with immune infiltration

**Fig. 1:**
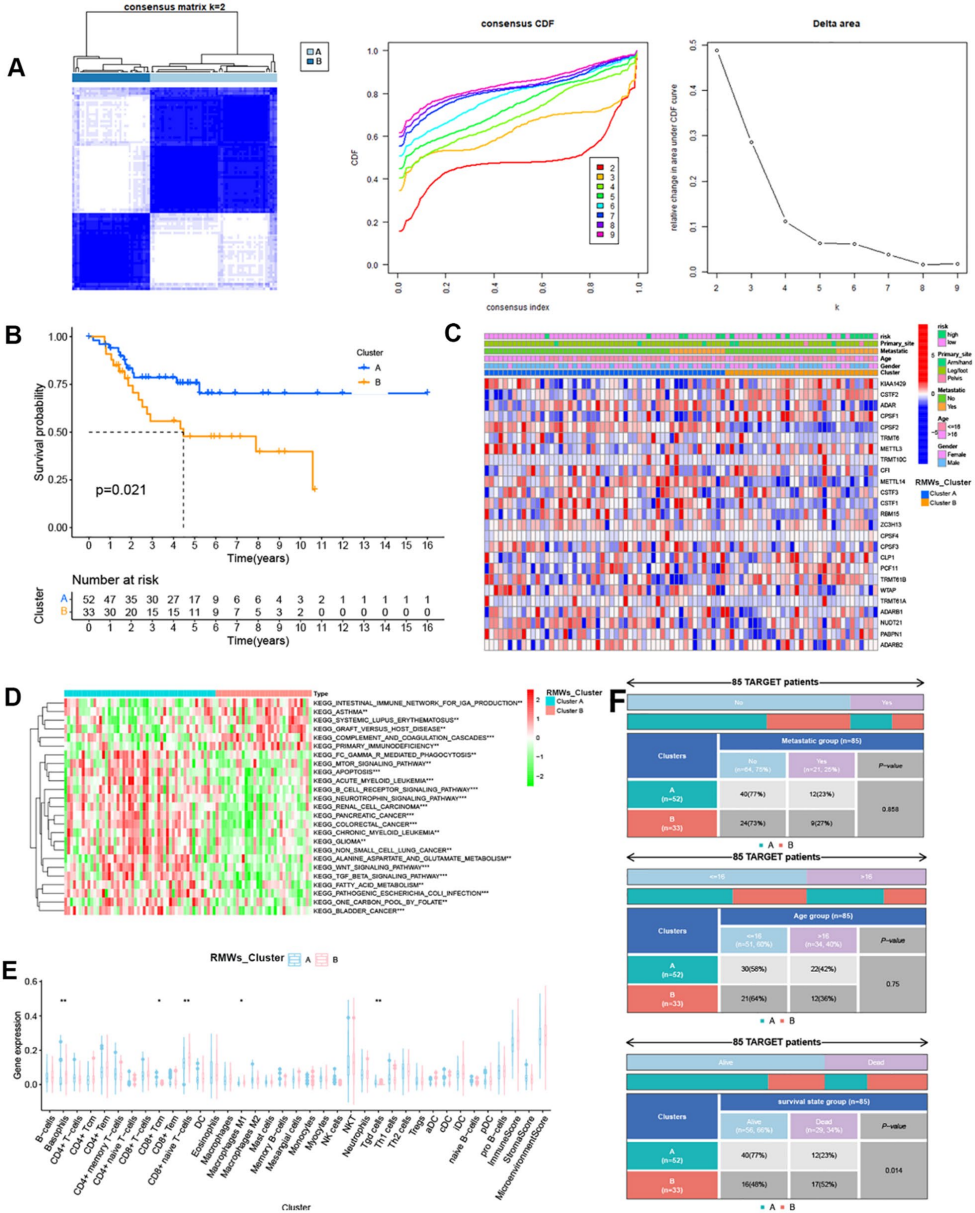
RMW patterns associated with immune infiltration. **A** Consensus clustering analysis. **B** The survival analysis of patients from two RMW patterns. **C** Heatmap of RMW expression. **D** Heatmap of differentially expressed signalling pathways in the two patterns. **E** Immune cell infiltration in two RMW patterns. **F** The relationship between RMW patterns and clinical characteristics

As noted in previous studies, a total of 26 RNA modification “writers” (RMWs, including 7 m^6^A “writers”, 4 m^1^A “writers”, 3 A to I “writers”, and 12 APA “writers”) were collected to analyse the prognostic role in OS. Twenty-six RMWs were observed in OS tissues from the target dataset and GSE21257 dataset, as shown in Additional file 1: Fig. S1A and B. Next, 26 RMWs were used for consensus clustering analysis, and 2 clusters were identified (Fig. 1A). The survival analysis showed that patients in Cluster B had poor overall survival (Fig. 1B). The heatmap revealed RMW expression and clinical characteristics in OS (Fig. 1C). GSVA and xCell analysis showed that Cluster B showed higher immune-related signals and immune cell infiltration (Fig. 1D and E). The chi-square test results showed that RMW patterns were associated with survival state and metastatic characteristics (Fig. 1F).

### DEGs patterns associated with immune infiltration

**Fig. 2:**
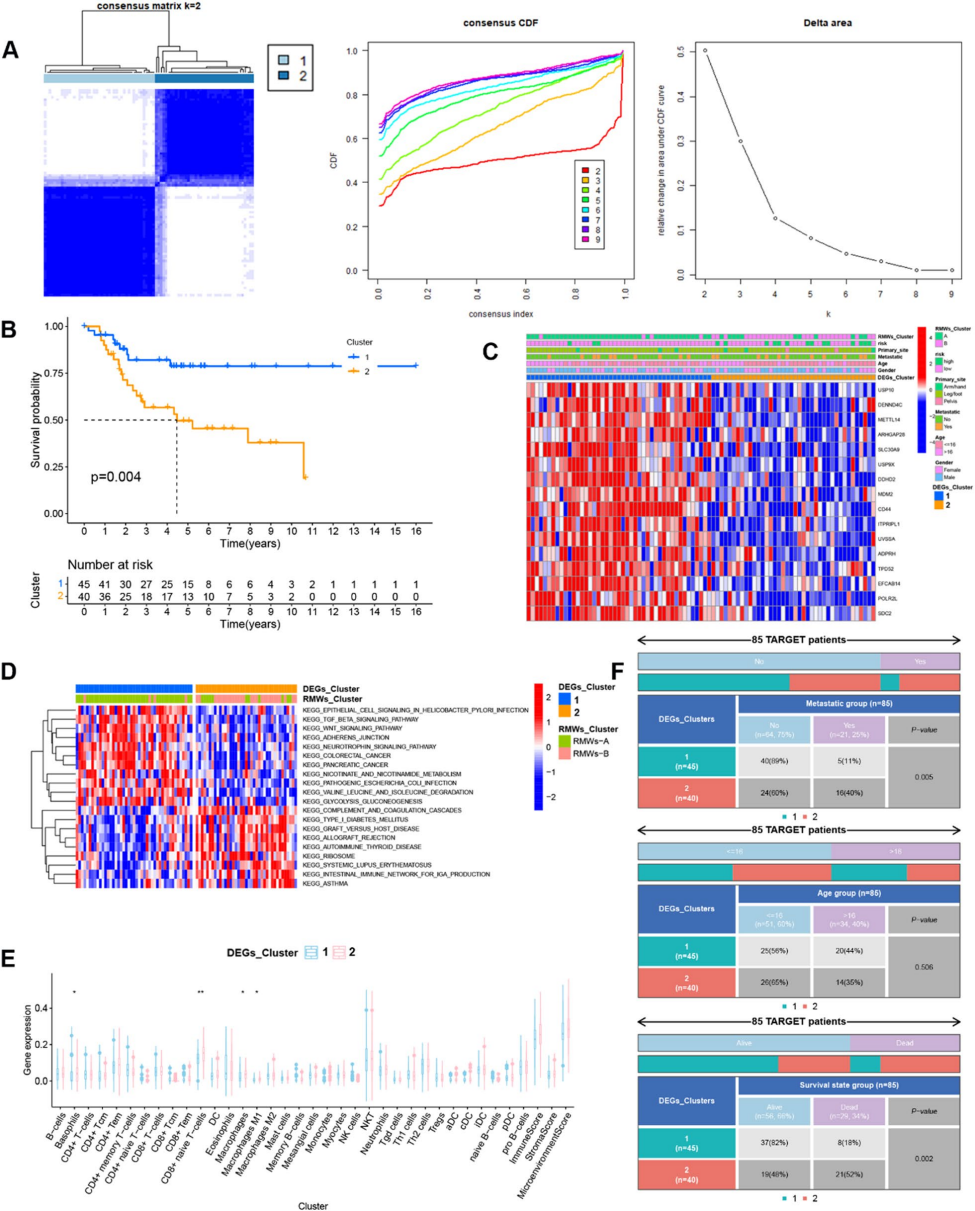
DEGs patterns associated with immune infiltration. **A** Consensus clustering analysis. **B** The survival analysis of patients from two DEG patterns. **C** Heatmap of DEG expression. **D** Heatmap of differentially expressed signalling pathways in the two patterns. **E** Immune cell infiltration in two DEG patterns. **F** The relationship between DEG patterns and clinical characteristics

To further describe the functional role of the RMW patterns above, 316 DEGs were identified in Cluster B compared to Cluster A. GO and KEGG enrichment analyses showed that the DEGs were enriched in cancer-related signalling pathways and apoptosis-, senescence- and immune-related pathways (Additional file 1: Fig. S1C). Next, 16 of 316 DEGs were associated with overall survival using Cox analysis. The 16 DEGs were used for consensus clustering analysis, and 2 DEG clusters were identified (Fig. 2A). The survival analysis showed that patients in DEG Cluster 2 had poor overall survival (Fig. 2B). The heatmap revealed the expression of the 16 risk-related DEGs and clinical characteristics in OS (Fig. 2C). We found that most patients in Cluster A were in Cluster 1, and most patients in Cluster B were in Cluster 2. Consistent with the RMW pattern, Cluster 2 showed higher immune-related signals and immune cell infiltration (Fig. 2D and E), and the DEG patterns were associated with survival state and metastatic characteristics (Fig. 2F).

### RMW-associated prognostic signature in OS

**Fig. 3:**
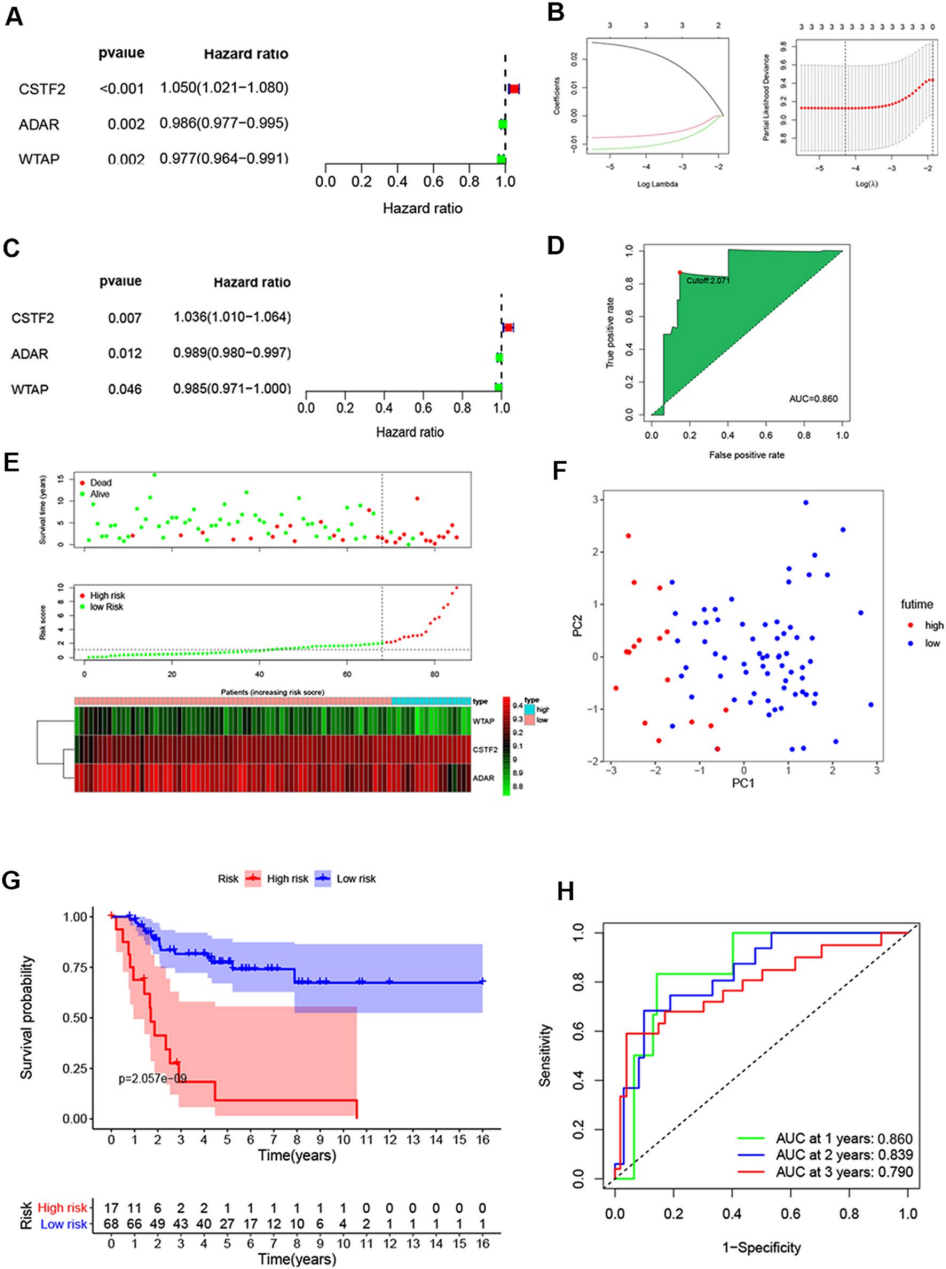
The RMW risk model in the Target dataset. **A** Univariate Cox regression models identified 3 RMWs associated with OS. **B** Lasso regression analysis of RMWs in OS. **C** A RMW prognostic model by multivariate Cox regression analysis. **D** ROC curve of the RMW signature in the Target datasets. **E** Risk score and survival status and heatmap of the RMW model for OS. **F** PCA of the high/low-risk groups. **G** Kaplan–Meier survival curve. **H** ROC curve analysis of the risk model for OS

To reveal the prognosis of RMWs in OS, univariate and multivariate Cox analyses were used to construct the RMW signature in OS using Target datasets. Three RMWs were related to the overall survival of OS patients (Fig. 3A). Lasso regression analysis and multivariate Cox regression analysis were used to construct the risk model (Fig. 3B). The risk score was calculated as follows: risk score = (0.03583 × CSTF2) + (-0.011308 × ADAR) + (-0.01465 × WTAP) (Fig. 3C). The AUC value of the RMW risk model was 0.860, and the best cut-off value was used for the low/high-risk group (Fig. 3D). The survival results revealed the poor prognosis of OS patients with high expression of CSTF2 and low expression of ADAR and WTAP in both TCGA and GEO datasets (Additional file 1: Fig. S2).

Seventeen patients and 68 patients were included in the high- and low-risk groups, respectively (Fig. 3E). The PCA demonstrated the markedly different distribution of the OS patients with high/low risk (Fig. 3F). OS patients with high risk showed a poorer prognosis than those with low risk (Fig. 3G). The AUC values of the RMWs were 0.860, 0.839, and 0.790 for survival times of 1 year, 2 years and 3 years, respectively, in the Target dataset (Fig. 3H). The results indicate that the model was convincing, specific, and sensitive.

### The relationship between RMWs signature and clinical characteristics

**Fig. 4:**
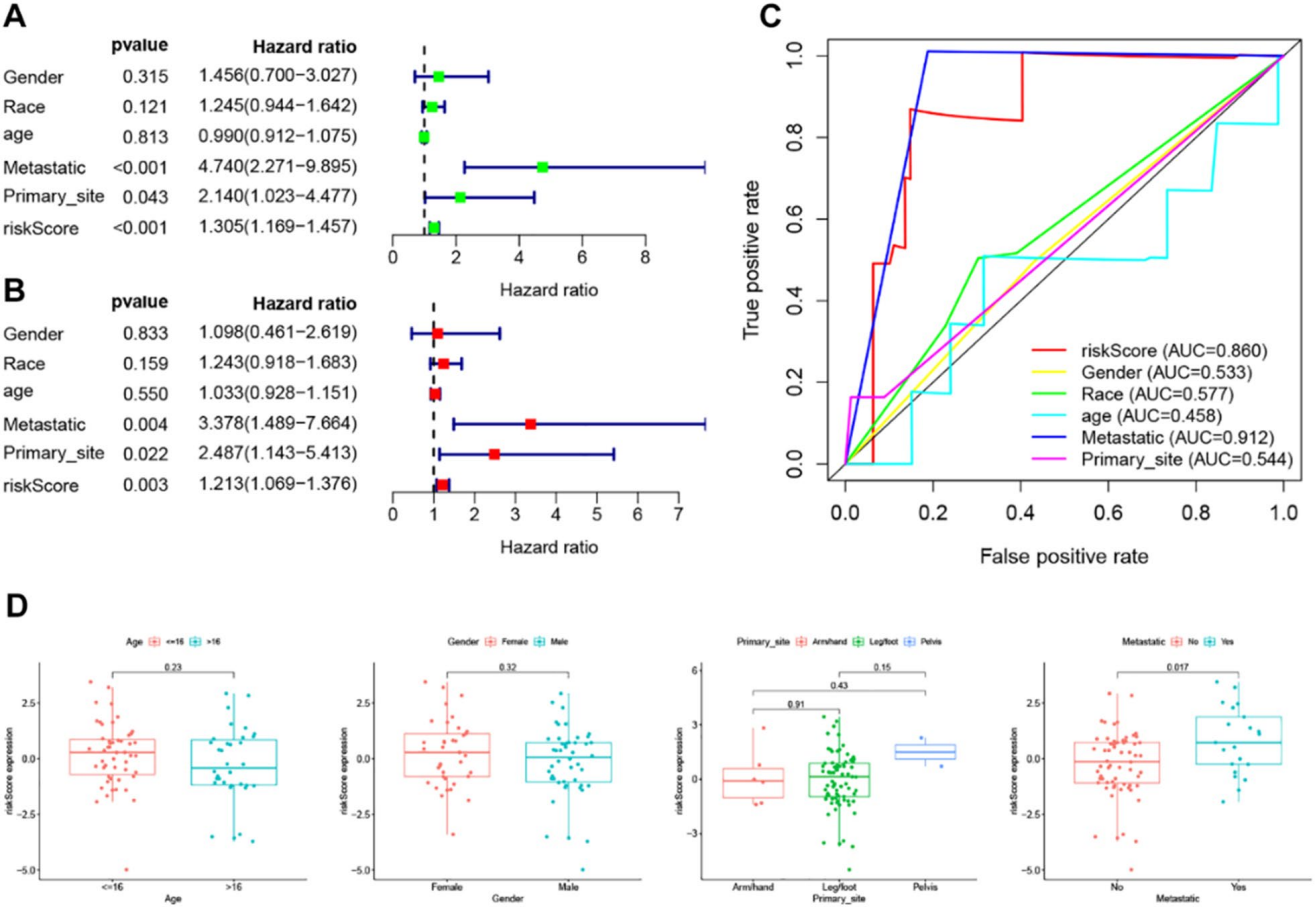
The relationships between clinical characteristics and the RMW signature in OS. **A**, **B** The independent prognostic factors of OS. **C** The ROC curve of the RMW signature and clinical characteristics of OS. **D** The relationship between the risk score and clinical characteristics

Next, we analysed the independent prognostic factor in OS. We found that Metastatic (*P* = 0.004), Primary_site (*P* = 0.022) and risk score (*P* = 0.003) were independent prognostic factors in the Target dataset (Fig. 4A and B). The ROC curve showed that the AUC values of Metastatic, RMWs, and Primary_site were 0.912, 0.860, and 0.544, respectively (Fig. 4C). We next analysed the relationship between the risk score and clinical characteristics. As shown in Fig. 4D, the risk score was higher in the metastatic group than in the nonmetastatic group. The relationships between risk RMWs and clinical characteristics in the target datasets are shown in Additional file 1: Fig. S3.

**Fig. 5:**
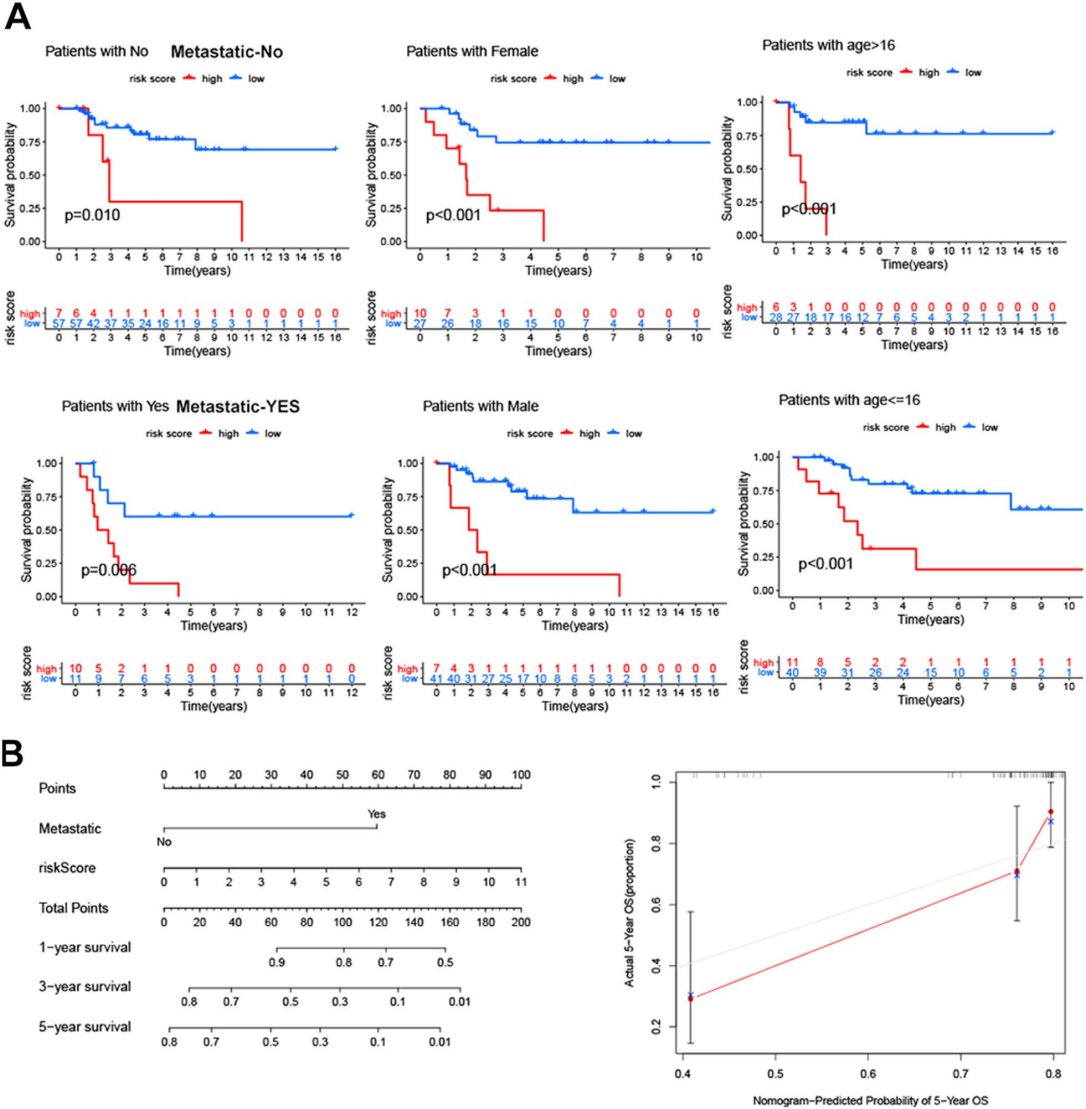
The RMW signature and clinical characteristics in OS. **A** The prognostic value of RMWs in different clinical subgroups. **B** The nomogram and calibration curve of OS

Next, we analysed survival in different clinical subgroups and low/high signatures. We found that a high-risk score was related to poor prognosis in age, metastasis and sex (Fig. 5A). Finally, the nomogram and calibration curves were applied to estimate the survival probabilities in OS (Fig. 5B), implying that the RMW signature provided an accurate prognosis of OS.

### Validation of the RMW signature in the GEO dataset

**Fig. 6:**
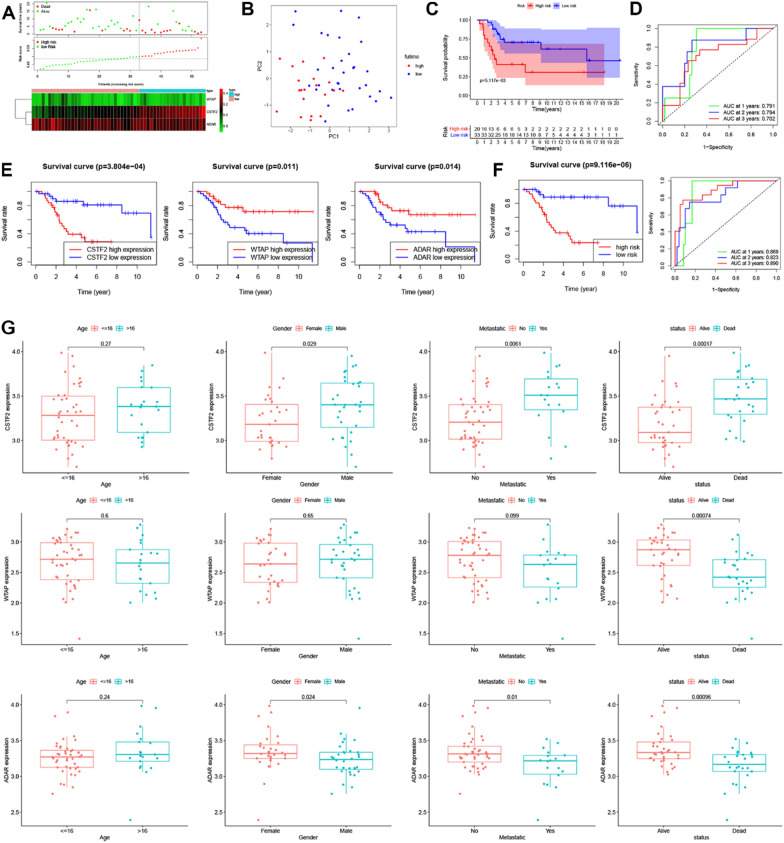
Validation of the RMW signature using the GSE21257 dataset and OS tissue detected by qPCR analysis. **A** Risk score and survival status and heatmap of the RMW model in OS. **B** PCA of the high/low-risk groups. **C** Kaplan–Meier survival curve. **D** ROC curve analysis of the risk model for OS. **E** Survival analysis of OS patients with differential expression of CSTF2, ADAR and WTAP. **F** Survival analysis of OS patients with high/low risk and the ROC analysis of the risk model. **G** The relationship between risk genes and clinical characteristics

We next validated the RMW signature in GSE21257 with 53 OS patients. The risk scores of OS patients in GSE21257 were calculated according to the formula results. Figure 6A shows 20 patients and 33 patients in the high- and low-risk groups, respectively. The PCA demonstrated the markedly different distribution of OS patients in the high/low-risk group (Fig. 6B). OS patients with high risk showed a poorer prognosis than those with low risk (Fig. 6C, p = 0.005117). The AUC values of the RMWs were 0.791, 0.794, and 0.702 for survival times of 1 year, 2 years and 3 years, respectively, in the GSE21257 dataset (Fig. 6D).

### RMW expression in OS tissue detected by qPCR analysis

To verify our previous results, we collected 63 OS tissues to detect RMW expression using qPCR. Table 2 shows the clinical information of the OS patients. As shown in Fig. 6E, the survival analysis demonstrated that OS patients with high expression of CSTF2 and OS patients with low expression of ADAR and WTAP showed poor prognoses. Moreover, OS patients with high risk showed poor prognoses, and the AUC values at 1, 2, and 3 years were 0.869, 0.823 and 0.890, respectively. (Fig. 6F). CSTF2, ADAR and WTAP expression was also related to OS metastasis. The CSTF2 expression level was significantly higher and ADAR and WTAP expression levels were significantly lower in OS patients who died (Fig. 6G).

**Table 2:** Clinical characteristics of OS patients from the Third Xiangya Hospital

	Patients	Percentage (%)
Age (years)		
≤ 16	42	66.66667
> 16	21	33.33333
Gender		
Male	32	50.79365
Female	31	49.20635
Metastatic		
No	45	71.42857
Yes	18	28.57143

### RMW signature-related immune infiltration in OS

**Fig. 7:**
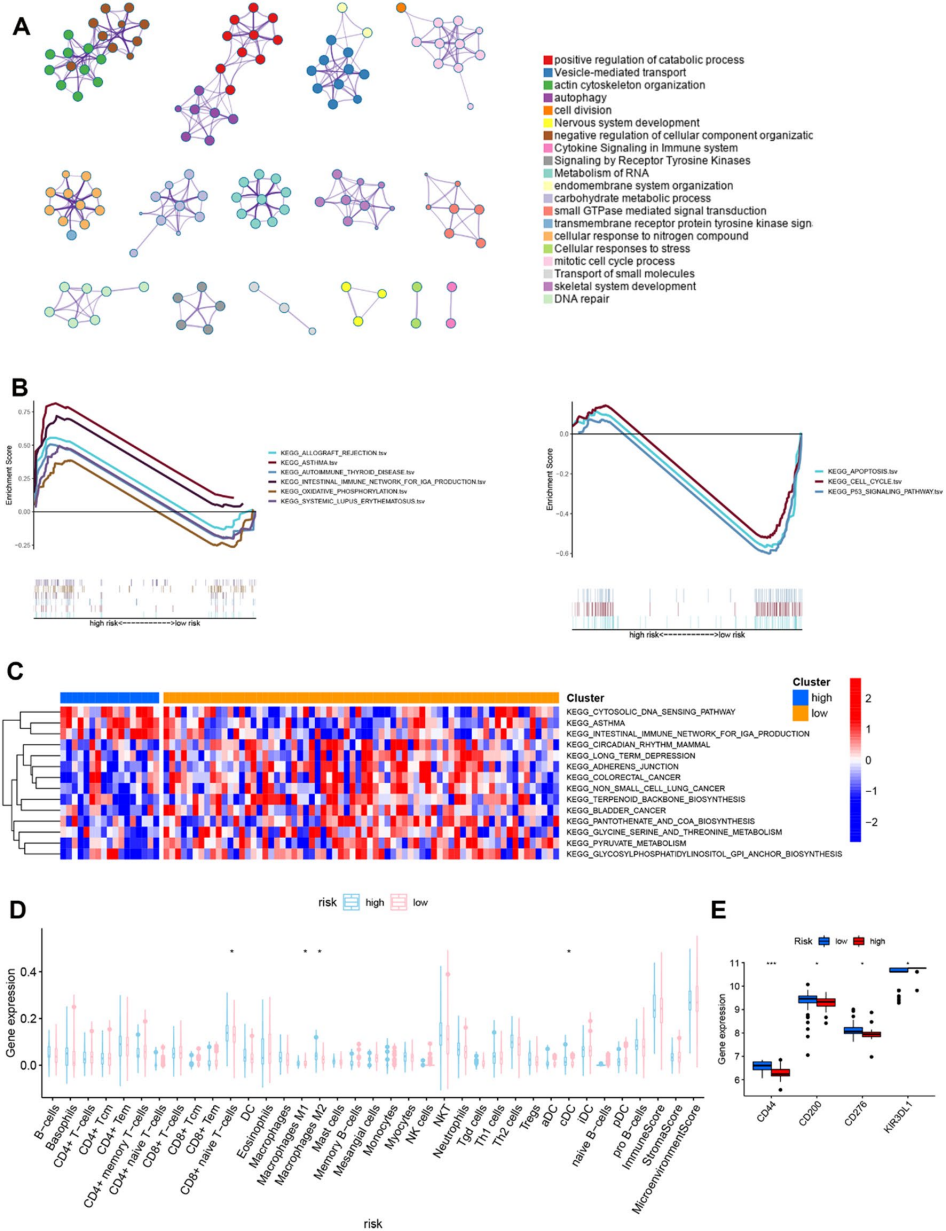
The RMW signature was related to immune infiltration in OS. **A** GO analysis revealed the risk signature-related pathways using Metascape. **B** GSEA was used to reveal the risk signature-related pathways in the Target dataset. C xCell analysis revealed immune infiltration in the high- and low-risk groups using the Target datasets. **D** ICB expression in the high- and low-risk groups. *P < 0.05

To further reveal the potential molecular mechanisms caused by RMW-mediated risk, we analysed the DEGs and related signalling pathways in high- and low-risk patients. A total of 1699 DEGs (494 upregulated and 1205 downregulated) were detected between the high- and low-risk groups. GO analysis showed that DEGs participate in the regulation of the catabolic process, cytokine signalling in the immune system, and cell cycle process (Fig. 7A). Next, GSEA and GSVA were used to reveal the risk signature-related pathways. As shown in Fig. 7B, the immune-related signalling pathway and cell cycle-related signalling pathway were regulated by high/low risk. The GSVA results showed that cancer- and metabolism-related and immune-related signalling pathways were differentially expressed in the low/high-risk groups (Fig. 7C). xCell was used to analyse the immune infiltration in OS, and 34 immune cells and 3 immune-related scores are shown in Fig. 7D. The results showed that the infiltration of CD8 naïve T cells, M1 macrophages, M2 macrophages and cDCs was evidently increased in the high-risk group. Moreover, Pearson analysis revealed a correlation between the risk score and immune cell infiltration (Additional file 1: Fig. S4). However, Fig. 7E shows that the immune checkpoint blockade (ICB) molecules CD44, CD200, CD276 and KIR3DL1 were differentially expressed between the high/low groups.

### Potential drugs for OS patients

**Fig. 8:**
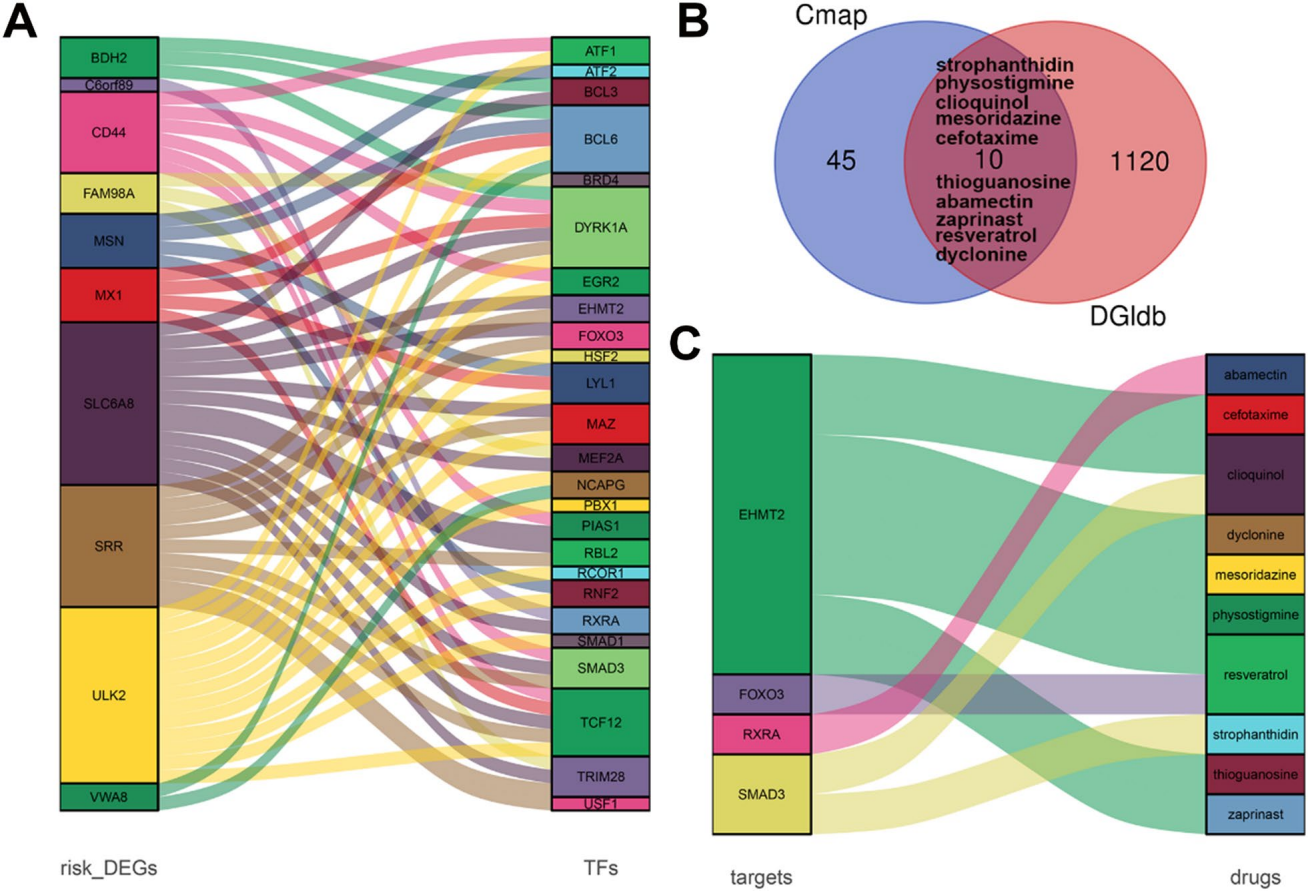
Potential drugs for OS patients. **A** The TF regulatory network in the high- and low-risk groups. **B** Drug prediction using the CMAP and DGldb databases. **C** The relationship between the drug and target genes in the DGldb database

To reveal the RMW-related hub genes, 12 prognosis-related genes were identified from 699 DEGs (Additional file 1: Table S1), and cancer-related transcription factors related to prognostic genes were identified, as shown in Additional file 1: Table S2 (R > 0.4 and p < 0.05). The 10 prognostic genes and 25 related transcription factor regulatory networks are shown in Fig. 8A. Then, the 35 hub genes were submitted to CMAP and DGldb for drug prediction. Fifty-five drugs were identified by the CMap database (Additional file 1: Table S3), and 1130 drugs were identified by the DGldb database. In total 10 drugs overlapped, including dyclonine, resveratrol, zaprinast, abamectin, thioguanosine, cefotaxime, mesoridazine, clioquinol, physostigmine and strophanthidin (Fig. 8B). The relationship between drugs and targets is described in Fig. 8C. Strophanthidin, a cardiac glycoside, was reported to be a promising anticancer agent by regulating the cell cycles of breast, lung and liver cancer cells [15]. It is also an immune cell activator that activates ROR T receptors [16]. Therefore, we selected strophanthidin for further functional analysis.

### Strophanthidin is an effective anti-OS drug in vitro and in vivo

**Fig. 9:**
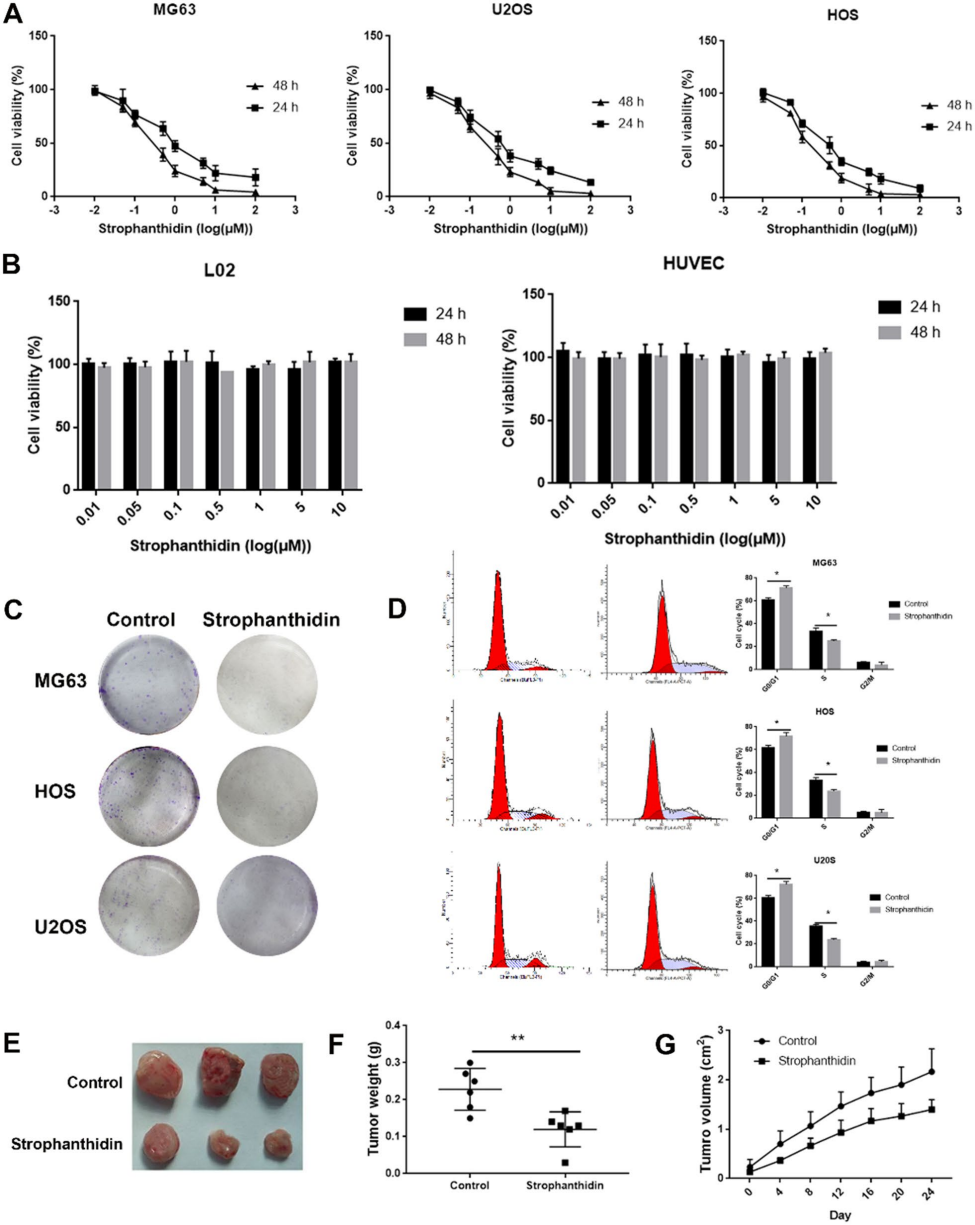
Strophanthidin is an effective anti-OS drug. **A** The MTT assay detected the effects of strophanthidin on OS cell viability. **B** The MTT assay detected the effects of strophanthidin on L02 and HUVEC viability. **C** A colony formation assay detected the effects of strophanthidin on OS cell proliferation. **D** Flow cytometry revealed the effects of strophanthidin on OS cell cycle arrest. **E–G** The effects of strophanthidin subcutaneous tumour formation in vivo

We next detected the effects of strophanthidin on cell proliferation in OS cells (MG63, HOS and U2OS). As shown in Fig. 9A, strophanthidin inhibited the proliferation ability of OS (MG63, HOS and U2OS) cells in a dose-dependent manner. The IC50 value of strophanthidin in HOS cells was much lower than those in MG63 and U2OS cells. However, 1 μM strophanthidin did not have significant effects on L02 cells or HUVECs (Fig. 9B). The colony assay also showed that 1 μM strophanthidin significantly reduced the number of colons in MG63, HOS and U2OS cells (Fig. 9C). We next analysed the role of strophanthidin in the OS cell cycle. As shown in Fig. 9D, strophanthidin (1 μM) treatment induced G1 arrest in OS cells. Furthermore, we detected the expression of cell cycle-related proteins in OS cells. To further reveal the inhibition of strophanthidin on OS growth, 0.5 mg/kg strophanthidin was used to treat OS mice. As shown in Fig. 9E-G, strophanthidin treatment reduced tumour growth in vivo.

## Discussion

OS is a commonly aggressive tumour in children and adolescents with poor survival rates. Recently, prognostic models for improving the prognosis of OS have attracted considerable attention. In this study, we constructed and verified the RMW patterns and prognostic signature in OS and shed light on strophanthidin as a novel therapeutic drug for OS patients.

RNA adenosine modification is the most common type of RNA modification and is involved in various pathophysiological processes, including tumorigenesis [17, 18]. m6A modification is an RNA modification with dynamic and reversible posttranscriptional characteristics that affects RNA processing, degradation and translation. Studies have shown that m6A modification levels are dysregulated in tumour tissues and are associated with the occurrence and progression of various tumours [19–22]. The “writers” play a key role in these RNA modifications, and some of the writers were reported as therapeutic targets of tumours. For example, METTL3, the m^6^A writer, is related to the prognosis and progression of acute myeloid leukaemia (AML), and METTL3 inhibition was reported as a therapeutic strategy for myeloid leukaemia [23]. A-to-I RNA editing is another abundant RNA modification event affecting adenosines in mammals, playing a critical role in the pathogenesis of various tumours [24]. Xu et al. revealed the role of A-to-I-edited miRNA cancer progression and highlighted the translational potential of edited miRNAs as a new class of cancer therapeutics [25]. Moreover, other adenine-related RNA modifications (m1A methylation and APA) have recently also emerged as key players in cancer pathophysiology by regulating the expression of cancer-related genes [26]. In OS, m^6^A modification of YAP is involved in the progression of OS [22]. The relationship between other adenine-related RNA modifications and OS remains unknown. RNA modification plays an important role in various biological processes through interactions with various “writers” (RMWs). Recent studies have focused on RNA modification signatures to evaluate the prognosis of various tumours. Our study explored RMW patterns and RNA modification DEG patterns that were associated with the survival of OS patients. Next, the RMW prognostic signature was constructed and identified as an independent prognostic factor for OS. In conclusion, these results indicated the important role of RMWs in OS. Although previous studies revealed several prognostic models (including ferroptosis-related gene signatures and autophagy-related prognosis models) for OS [27–30], this is the first study of the RWM-related prognosis model and RMP patterns for OS. Moreover, the ROC of the RMW model was considerably increased compared with that of the ferroptosis-related gene signature (ROC = 8.06) and autophagy-related prognosis model (ROC = 8.38). These results indicated that the RMW signature is a more effective prognostic model for OS.

The immune microenvironment (TME) is related to the prognosis of various cancers, including OS [31–33]. The recruitment of stromal cells was reported to be associated with a poor patient prognosis. Infiltrating myeloid cells are common and important components of the TME, and they are reported to drive OS progression [34]. Tumour-infiltrating myeloid cells include monocytes, dendritic cells (DCs), tumour-associated macrophages (TAMs), and neutrophils. In OS lesions, monocytes and macrophages are the most common myeloid cells, and DCs account for < 5% of myeloid cells [35]. M2 TAMs are anti-inflammatory macrophages and are often associated with a worse prognosis [36]. M1 TAMs are antitumor immune cells that express proinflammatory cytokines. Anti-PD-1 is an effective therapeutic strategy for OS due to the regulation of the infiltration of M1 and M2 macrophages in tumour tissue [37]. Recently, DCs were identified as therapeutic targets of immunotherapy due to their powerful antigen-presenting features. Zhou et al. showed that the infiltration of CD1c^+^ DCs was significantly increased in metastatic OS [35]. In this study, we used xCell to reveal the correlations between the risk signature and immune infiltration. We found that M1 macrophages, M2 macrophages and cDCs were significantly increased in OS patients at high risk. Recent studies shed new light on the regulation of RNA modification on the immune system [38]. Furthermore, RNA adenosine modifications are associated with immunoregulation in tumour tissues [38]. METTL3-mediated m6A modification promotes tumour growth and metastasis by macrophage reprogramming [39]. METTL3-mediated m6A modification also affects resistance to chemotherapy by regulating M2-TAM infiltration [40]. METTL3-mediated m6A methylation regulates dendritic cell activation [41]. These results highlighted that RNA adenosine modifications might affect the prognosis of OS partly by regulating the immune microenvironment.

DGIdb and CMap, drug prediction databases, are widely used to screen drugs that can regulate certain target genes. In this study, we utilized these two databases to obtain the possible RMW-related drugs for OS. Ten drugs were identified, including dyclonine, resveratrol, zaprinast, abamectin, thioguanosine, cefotaxime, mesoridazine, clioquinol, physostigmine and strophanthidin. Dyclonine, an ALDH3A1 inhibitor, is widely used as a topical antipruritic agent [42]. Recent studies reported that dyclonine enhances MG132-induced cell cytotoxicity in breast cancer [43]. Resveratrol, an oestrogenic compound, was reported as a treatment for osteoporosis with anti-inflammatory and antioxidant properties [44]. Recent studies also revealed the therapeutic potential of resveratrol on various tumours, including OS [45–47]. Zaprinast, a synthetic GPR35 agonist, was reported to rescue OVX-induced bone [48]. It also exhibits antitumor activity in colon cancer, lung cancer, and melanoma [49–51]. Abamectin, an anthelmintic agent in animals, induces oxidative stress in cerebral and hepatic tissues [52]. Thioguanosine has antitumor activities against hepatocellular carcinoma [53]. Mesoridazine, an antipsychotic drug, has cardiac conduction side effects with fatal consequences in patients [54]. Clioquinol has been reported as an antitumor drug for OS [54]. Physostigmine has been reported to repress pancreatic cancer growth with anticholinergic toxicity [55]. Strophanthidin, a cardiac glycoside, was reported to be a promising anticancer agent by regulating the cell cycles of breast, lung and liver cancer cells [15]. It is also an immune cell activator that activates ROR T receptors [16]. Therefore, we selected strophanthidin for further functional analysis. In this study, in vivo and in vitro experiments showed that strophanthidin elicited antitumor activity against OS by repressing cell proliferation and the cell cycle. As shown in Fig. 8, Smad3 was predicted to be the target of strophanthidin. Smad3 is a key mediator of TGF-β signalling, an important signalling pathway in tumorigenesis [56]. Smad3 was also reported to be involved in the regulation of the cell cycle via the induction of CDK inhibitors [57, 58]. Based on the above research, we hypothesize that strophanthidin regulates the cell cycle of OS cells by targeting Smad3.

## Conclusions

In summary, we analysed RNA modification “writers” (RMWs) in OS and revealed RMW patterns and signatures associated with the cell cycle and immune infiltration. Strophanthidin was identified and verified as a candidate therapy for OS by repressing the growth and cell cycle of OS cells.

## Supplementary Information

Additional file 1: **Figure S1. A,** Heatmap of RMWs in OS tissue from the Target dataset and GSE21257 dataset. **B, **GO and KEGG analyses of DEGs. **Figure S2.** Survival analysis of OS patients with differential expression of CSTF2, ADAR and WTAP in both TCGA and GEO datasets. **Figure S3.** The correlation between risk RMW expression and clinical characteristics. **Figure S4. **The relationship between the risk score and immune infiltration was analysed using Pearson’s correlation analysis. **Table S1. **Univariate Cox analysis revealed prognosis-related DEGs in the high- and low-risk groups. **Table S2. **The relationship between TF and prognosis-related DEGs. **Table S3.** Drug prediction using the CMap database.

## Data Availability

The data used to support the findings of this study are available either online or from the corresponding author upon request.
