# Supplementary material for: RNA adenosine modifications related to prognosis and immune infiltration in osteosarcoma
Source: J Transl Med. 2022 May 14;20:228. doi: 10.1186/s12967-022-03415-6 (PMC9107650; doi:10.1186/s12967-022-03415-6)
Supplement: Supplementary file 1 — Additional file 1: Figure S1. A, Heatmap of RMWs in OS tissue from the Target dataset and GSE21257 dataset. B, GO and KEGG analyses of DEGs. Figure S2. Survival analysis of OS patients with differential expression of CSTF2, ADAR and WTAP in both TCGA and GEO datasets. Figure S3. The correlation between risk RMW expression and clinical characteristics. Figure S4. The relationship between the risk score and immune infiltration was analysed using Pearson’s correlation analysis. Table S1. Univariate Cox analysis revealed prognosis-related DEGs in the high- and low-risk groups. Table S2. The relationship between TF and prognosis-related DEGs. Table S3. Drug prediction using the CMap database. [file 12967_2022_3415_MOESM1_ESM.docx]

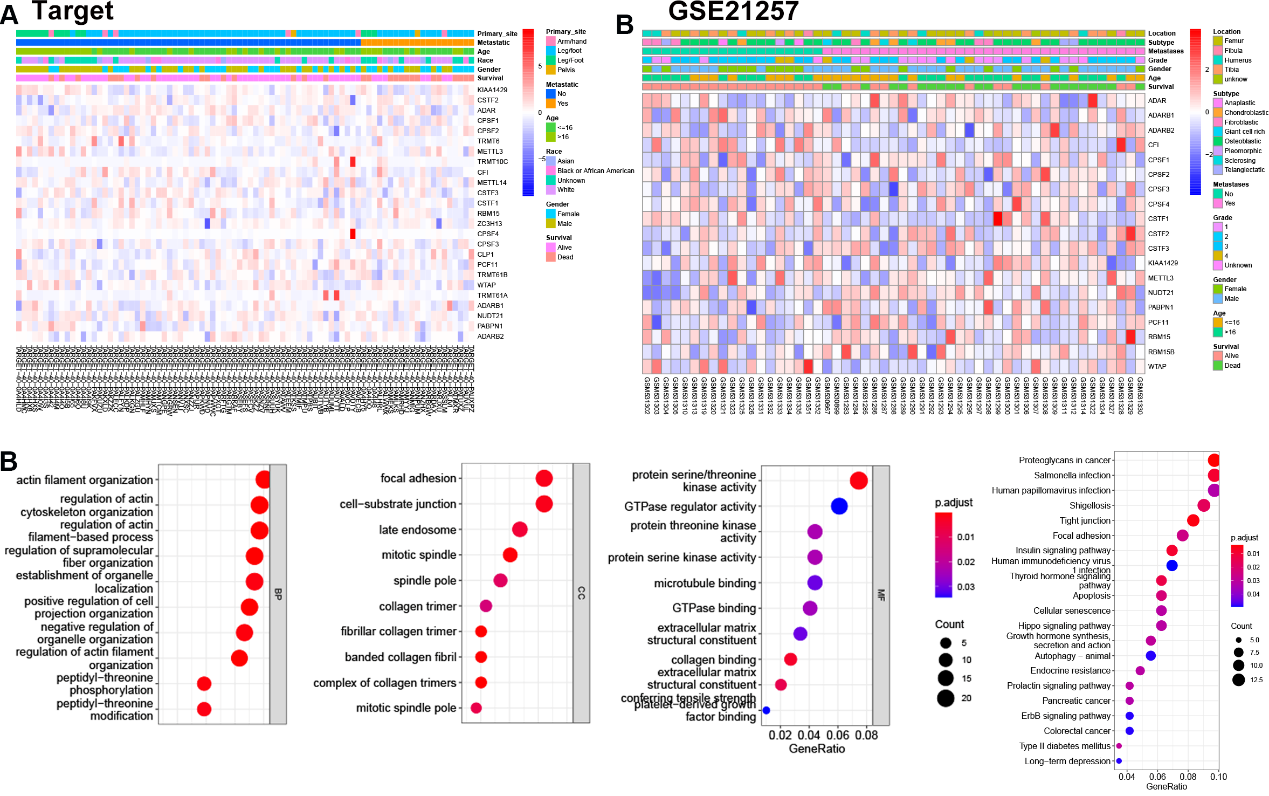


**Figure S1. A,** Heatmap of RMWs in OS tissue from the Target dataset and GSE21257 dataset. **B,** GO and KEGG analyses of DEGs.


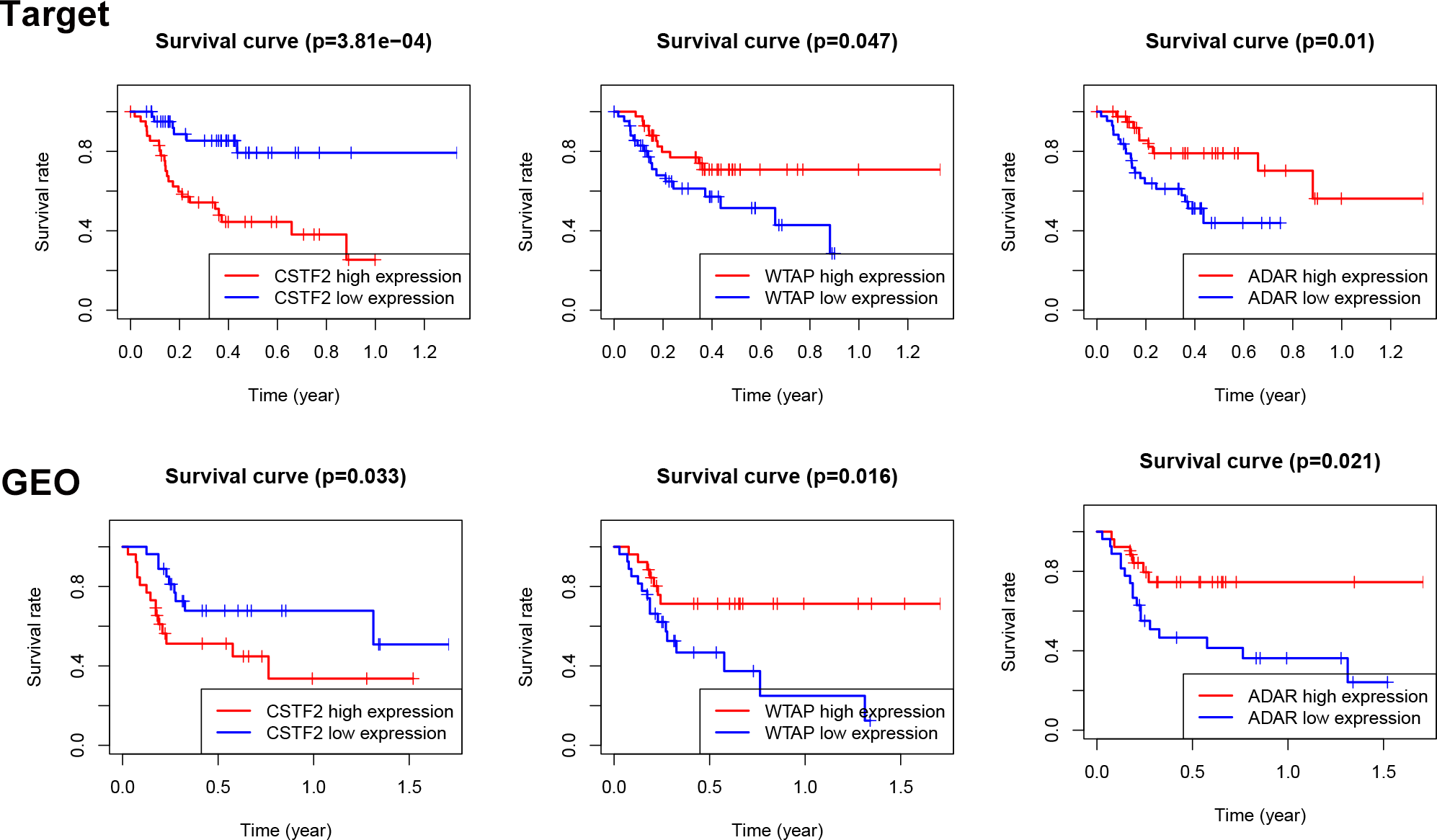


**Figure S2.** Survival analysis of OS patients with differential expression of CSTF2, ADAR and WTAP in both TCGA and GEO datasets.


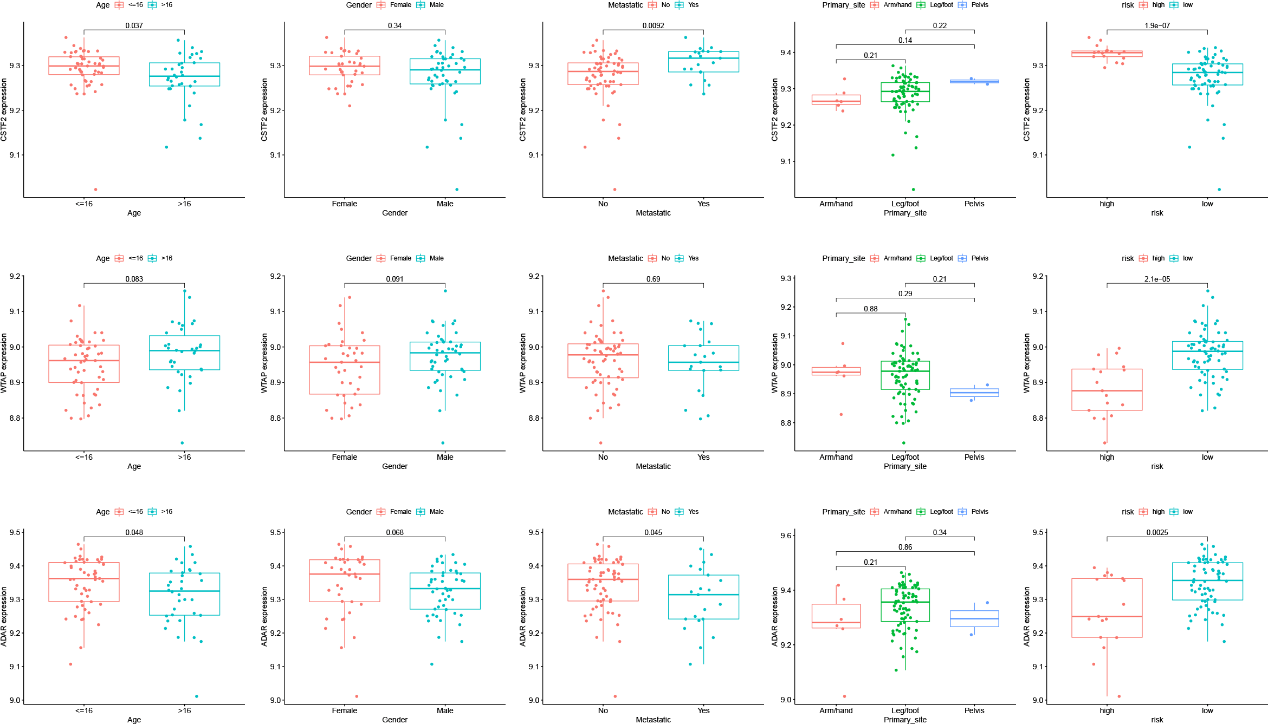


**Figure S3.** The correlation between risk RMW expression and clinical characteristics.


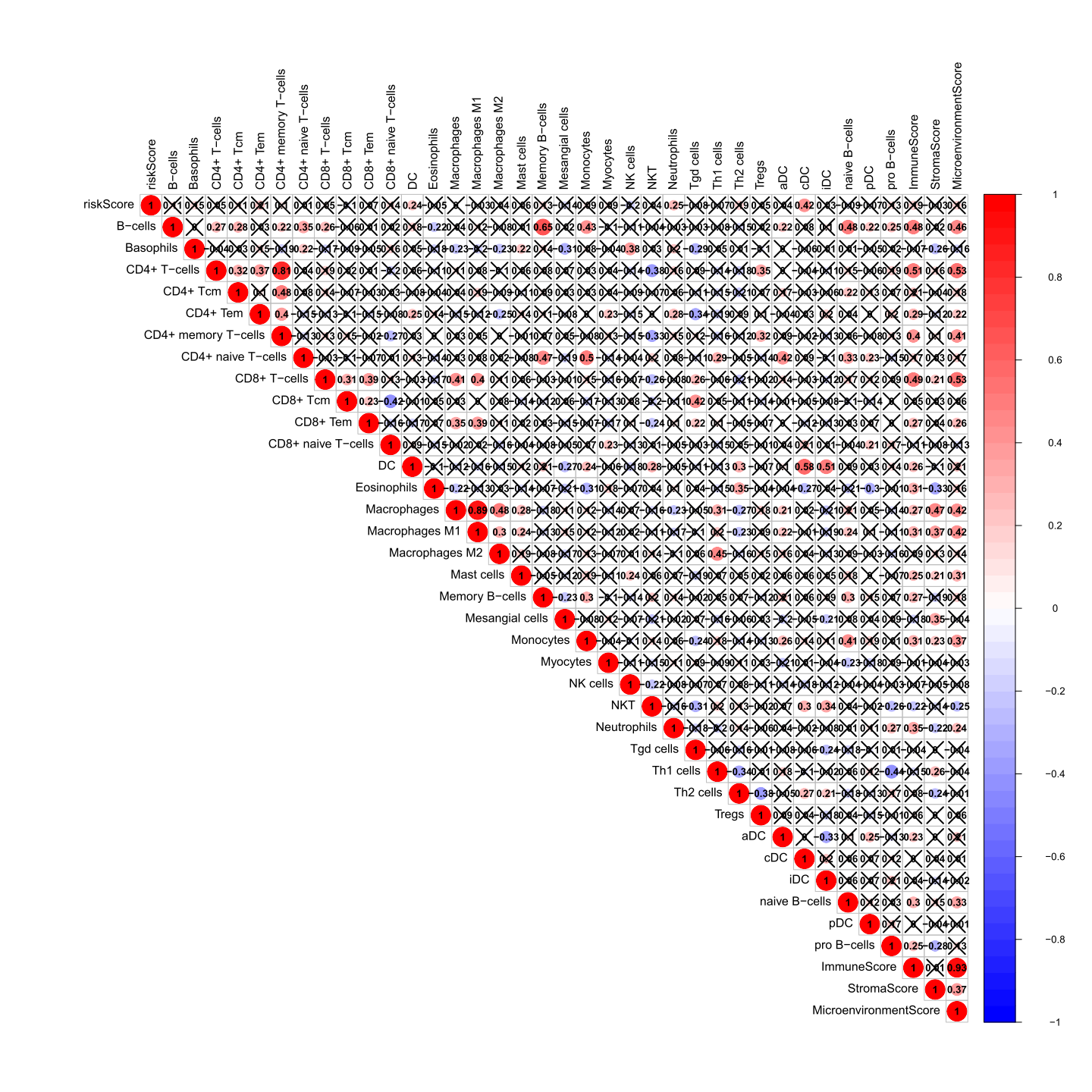
**Figure S4.** The relationship between the risk score and immune infiltration was analysed using Pearson’s correlation analysis.

**Table S1.** Univariate Cox analysis revealed prognosis-related DEGs in the high- and low-risk groups

| id | HR | HR.95L | HR.95H | p value |
| --- | --- | --- | --- | --- |
| VWA8 | 0.997 | 0.995 | 0.999 | 0.001 |
| MSN | 0.999 | 0.998 | 1.000 | 0.045 |
| ALG13 | 0.961 | 0.927 | 0.996 | 0.027 |
| CD44 | 0.971 | 0.949 | 0.993 | 0.011 |
| ULK2 | 0.995 | 0.991 | 0.999 | 0.014 |
| SRR | 0.994 | 0.987 | 1.000 | 0.039 |
| BDH2 | 1.029 | 1.015 | 1.043 | 0.000 |
| MX1 | 0.990 | 0.982 | 0.998 | 0.011 |
| FAM98A | 0.937 | 0.892 | 0.985 | 0.010 |
| SLC6A8 | 0.981 | 0.967 | 0.995 | 0.010 |
| MRPL35 | 0.998 | 0.996 | 1.000 | 0.012 |
| C6orf89 | 0.999 | 0.998 | 1.000 | 0.028 |

**Table S2.** The relationship between TF and prognosis-related DEGs

| Prognosis_related_DEGs | TFs | cor | p value | FDR |
| --- | --- | --- | --- | --- |
| VWA8 | BCL6 | 0.419189 | 6.52E-05 | 0.000603 |
| VWA8 | NCAPG | 0.458489 | 1.02E-05 | 0.000146 |
| MSN | ATF2 | 0.448992 | 1.63E-05 | 0.000201 |
| MSN | BCL6 | 0.457092 | 1.09E-05 | 0.000147 |
| MSN | LYL1 | -0.46368 | 7.85E-06 | 0.000116 |
| MSN | RNF2 | 0.423834 | 5.30E-05 | 0.000535 |
| CD44 | EGR2 | -0.40972 | 9.87E-05 | 0.000842 |
| CD44 | TCF12 | 0.556529 | 3.19E-08 | 1.42E-06 |
| CD44 | PIAS1 | 0.471934 | 5.13E-06 | 8.13E-05 |
| CD44 | ATF1 | 0.504197 | 8.67E-07 | 2.41E-05 |
| CD44 | SMAD3 | 0.512703 | 5.26E-07 | 1.67E-05 |
| CD44 | DYRK1A | 0.467404 | 6.49E-06 | 9.93E-05 |
| ULK2 | EGR2 | -0.42222 | 5.70E-05 | 0.00054 |
| ULK2 | MAZ | -0.41396 | 8.21E-05 | 0.000715 |
| ULK2 | BCL6 | 0.499934 | 1.11E-06 | 2.73E-05 |
| ULK2 | TCF12 | 0.663043 | 4.73E-12 | 2.10E-09 |
| ULK2 | RCOR1 | 0.417928 | 6.90E-05 | 0.000625 |
| ULK2 | HSF2 | 0.406827 | 0.000112 | 0.000902 |
| ULK2 | PBX1 | 0.478319 | 3.66E-06 | 6.25E-05 |
| ULK2 | SMAD1 | -0.49687 | 1.32E-06 | 3.08E-05 |
| ULK2 | ATF1 | 0.588517 | 3.15E-09 | 3.50E-07 |
| ULK2 | LYL1 | -0.42723 | 4.55E-05 | 0.000491 |
| ULK2 | RNF2 | 0.556478 | 3.20E-08 | 1.42E-06 |
| ULK2 | DYRK1A | 0.527144 | 2.18E-07 | 8.81E-06 |
| ULK2 | NCAPG | 0.520085 | 3.37E-07 | 1.15E-05 |
| SRR | FOXO3 | -0.42675 | 4.65E-05 | 0.000491 |
| SRR | RBL2 | 0.476599 | 4.01E-06 | 6.59E-05 |
| SRR | MAZ | -0.4068 | 0.000112 | 0.000902 |
| SRR | TCF12 | 0.444992 | 1.98E-05 | 0.000231 |
| SRR | TRIM28 | 0.403873 | 0.000127 | 0.000988 |
| SRR | SMAD3 | 0.478365 | 3.65E-06 | 6.25E-05 |
| SRR | USF1 | -0.60625 | 7.82E-10 | 1.46E-07 |
| SRR | DYRK1A | 0.560264 | 2.47E-08 | 1.37E-06 |
| SRR | EHMT2 | -0.58447 | 4.29E-09 | 3.81E-07 |
| BDH2 | BCL3 | -0.42849 | 4.29E-05 | 0.000477 |
| BDH2 | BCL6 | -0.45384 | 1.29E-05 | 0.000168 |
| BDH2 | DYRK1A | -0.42425 | 5.20E-05 | 0.000535 |
| MX1 | BCL6 | 0.491688 | 1.76E-06 | 3.92E-05 |
| MX1 | TCF12 | 0.457345 | 1.08E-05 | 0.000147 |
| MX1 | LYL1 | -0.50236 | 9.64E-07 | 2.52E-05 |
| MX1 | DYRK1A | 0.414562 | 8.00E-05 | 0.00071 |
| FAM98A | TRIM28 | 0.60333 | 9.89E-10 | 1.46E-07 |
| FAM98A | MEF2A | 0.432182 | 3.62E-05 | 0.000412 |
| FAM98A | BRD4 | 0.575637 | 8.25E-09 | 6.11E-07 |
| SLC6A8 | FOXO3 | -0.45309 | 1.34E-05 | 0.000169 |
| SLC6A8 | RBL2 | 0.482701 | 2.89E-06 | 5.83E-05 |
| SLC6A8 | MAZ | -0.40743 | 0.000109 | 0.000902 |
| SLC6A8 | BCL3 | 0.40382 | 0.000127 | 0.000988 |
| SLC6A8 | TCF12 | 0.480148 | 3.32E-06 | 6.25E-05 |
| SLC6A8 | TRIM28 | 0.487543 | 2.22E-06 | 4.69E-05 |
| SLC6A8 | PIAS1 | 0.422171 | 5.71E-05 | 0.00054 |
| SLC6A8 | MEF2A | 0.422761 | 5.56E-05 | 0.00054 |
| SLC6A8 | SMAD3 | 0.479062 | 3.52E-06 | 6.25E-05 |
| SLC6A8 | RXRA | 0.504798 | 8.37E-07 | 2.41E-05 |
| SLC6A8 | DYRK1A | 0.448412 | 1.68E-05 | 0.000201 |
| SLC6A8 | EHMT2 | -0.57092 | 1.16E-08 | 7.37E-07 |
| C6orf89 | RXRA | 0.521907 | 3.02E-07 | 1.12E-05 |

**Table S3.** Drug prediction using the CMap database.

| cmap name | mean | n | enrichment | p | specificity | percent nonnull |
| --- | --- | --- | --- | --- | --- | --- |
| lanatoside C | -0.531 | 6 | -0.81 | 0.00014 | 0.0213 | 100 |
| zaprinast | -0.613 | 4 | -0.888 | 0.00036 | 0 | 100 |
| strophanthidin | -0.617 | 4 | -0.884 | 0.00042 | 0 | 100 |
| atractyloside | 0.5 | 5 | 0.81 | 0.0006 | 0.0111 | 100 |
| helveticoside | -0.512 | 6 | -0.739 | 0.00066 | 0.013 | 83 |
| Gly-His-Lys | -0.577 | 3 | -0.917 | 0.00096 | 0.0149 | 100 |
| 16-phenyltetranorprostaglandin E2 | -0.569 | 4 | -0.848 | 0.00097 | 0.0119 | 100 |
| benfotiamine | -0.294 | 5 | -0.739 | 0.00246 | 0 | 60 |
| mephenesin | -0.391 | 5 | -0.73 | 0.00302 | 0.0117 | 80 |
| betulin | 0.617 | 3 | 0.883 | 0.0032 | 0.0063 | 100 |
| propantheline bromide | 0.49 | 4 | 0.796 | 0.00332 | 0 | 100 |
| karakoline | -0.417 | 6 | -0.654 | 0.00471 | 0 | 83 |
| atropine oxide | -0.351 | 5 | -0.704 | 0.00495 | 0.0063 | 60 |
| laudanosine | -0.361 | 4 | -0.777 | 0.00511 | 0 | 50 |
| resveratrol | 0.389 | 9 | 0.535 | 0.0057 | 0.2941 | 66 |
| physostigmine | 0.335 | 4 | 0.76 | 0.00627 | 0 | 75 |
| kinetin | -0.397 | 4 | -0.731 | 0.01052 | 0 | 75 |
| brinzolamide | -0.33 | 4 | -0.727 | 0.01128 | 0.0216 | 75 |
| albendazole | -0.55 | 3 | -0.821 | 0.0114 | 0.0286 | 100 |
| gramine | -0.268 | 4 | -0.718 | 0.01281 | 0.0328 | 50 |
| fenbufen | 0.349 | 6 | 0.601 | 0.01283 | 0 | 66 |
| tribenoside | 0.294 | 4 | 0.718 | 0.01297 | 0.0318 | 50 |
| ifosfamide | -0.495 | 3 | -0.81 | 0.0135 | 0.0182 | 100 |
| mebendazole | -0.366 | 5 | -0.644 | 0.0142 | 0.1165 | 80 |
| ivermectin | 0.244 | 5 | 0.645 | 0.01574 | 0.1129 | 80 |
| amodiaquine | 0.305 | 4 | 0.7 | 0.01667 | 0.0125 | 50 |
| cefotaxime | 0.286 | 5 | 0.64 | 0.0172 | 0 | 80 |
| latamoxef | 0.465 | 3 | 0.795 | 0.01747 | 0.029 | 100 |
| clioquinol | 0.408 | 5 | 0.637 | 0.01788 | 0.1518 | 80 |
| thiamine | 0.441 | 3 | 0.79 | 0.01867 | 0.0403 | 100 |
| trazodone | 0.571 | 3 | 0.788 | 0.01921 | 0.0896 | 100 |
| aminohippuric acid | -0.297 | 4 | -0.688 | 0.02037 | 0.0556 | 50 |
| acebutolol | 0.287 | 5 | 0.622 | 0.02251 | 0.0278 | 60 |
| lincomycin | 0.458 | 3 | 0.776 | 0.02269 | 0.0079 | 100 |
| dantrolene | 0.207 | 6 | 0.569 | 0.0237 | 0.0513 | 50 |
| dyclonine | -0.312 | 4 | -0.668 | 0.02727 | 0.0221 | 50 |
| mefexamide | 0.258 | 4 | 0.666 | 0.02787 | 0.0245 | 50 |
| pralidoxime | 0.397 | 4 | 0.657 | 0.03201 | 0.0638 | 75 |
| esculin | 0.297 | 4 | 0.657 | 0.03203 | 0 | 75 |
| nisoxetine | -0.286 | 4 | -0.654 | 0.03322 | 0.0366 | 50 |
| cotinine | 0.292 | 6 | 0.546 | 0.03369 | 0.0455 | 50 |
| alfadolone | 0.29 | 3 | 0.74 | 0.03413 | 0.0235 | 66 |
| Y-27632 | -0.47 | 2 | -0.869 | 0.03428 | 0.0492 | 100 |
| minaprine | 0.361 | 5 | 0.59 | 0.03565 | 0.02 | 80 |
| isocarboxazid | 0.267 | 5 | 0.587 | 0.03687 | 0.0592 | 60 |
| abamectin | 0.403 | 4 | 0.646 | 0.0374 | 0.04 | 75 |
| betahistine | -0.408 | 4 | -0.646 | 0.03756 | 0.08 | 75 |
| etifenin | -0.475 | 4 | -0.645 | 0.03804 | 0.0345 | 75 |
| mesoridazine | -0.273 | 4 | -0.641 | 0.03965 | 0.0546 | 50 |
| hydroflumethiazide | -0.173 | 5 | -0.574 | 0.04205 | 0.0567 | 60 |
| gibberellic acid | -0.422 | 4 | -0.635 | 0.04297 | 0.0642 | 75 |
| atropine methonitrate | -0.338 | 3 | -0.723 | 0.04383 | 0.0383 | 66 |
| amprolium | 0.313 | 5 | 0.572 | 0.0458 | 0.0385 | 80 |
| thioguanosine | 0.464 | 4 | 0.63 | 0.04683 | 0.2588 | 75 |
| ellipticine | 0.447 | 4 | 0.628 | 0.04798 | 0.3039 | 75 |
